# Development of a novel dental shade determination application

**DOI:** 10.34172/joddd.2020.011

**Published:** 2020

**Authors:** Les Kalman

**Affiliations:** ^1^Restorative Dentistry Chair, Dental Outreach Schulich School of Medicine & Dentistry, Western University, 1151 Richmond Street, N6A-3K7, London, Ontario, Canada

**Keywords:** dental shade, shade determination, digital dentistry, medical device, restorative dentistry, prosthodontics

## Abstract

Determining the shade of dental materials is a challenging requirement for the restorative dentist. Improper shade selection is the second cause for laboratory remakes, resulting in inefficiency and additional cost, and unnecessary stress for the clinician and patient. The process of shade selection is somewhat subjective, with no consensus on the protocol. This research investigation aimed to develop a novel software application to provide an accurate, objective, and systematic approach to shade determination for teeth, soft tissues, and dental materials. An IOS software application was developed, termed Smile Shade, to facilitate a simple approach for dental shade determination. Smile Shade functions on a high-dynamic-range microcolor sensor with automatic temperature control and inter-device repeatability of <1.0 ∆E. The determination of shade is completed through the evaluation of color based on CMYK, RGB, and LAB, which are different techniques of storing colors. Further research is underway to compare this novel application to the traditional shade tab approach commonly practiced at most dental schools.

## Introduction

Determining the shade of dental materials is a challenging requirement for the restorative dentist.^1^ From simple tooth-colored fillings to ceramic crowns, the correct shade needs to be determined and communicated to the dental laboratory for the successful delivery of treatment.^2^ Based on the numerous variables associated with shade selection, such as the light source, the tooth, the eye, and retinal fatigue, it proves to be challenging even for the experienced clinician.^2,3^ Moreover, shade selection presents a difficult endeavor for the dental student,^4^ with a success rate of 49%.^5^ Improper shade selection is the second cause for laboratory remakes,^6^ resulting in inefficiency and additional cost, and unnecessary stress for the clinician and patient.

**Figure 1:**
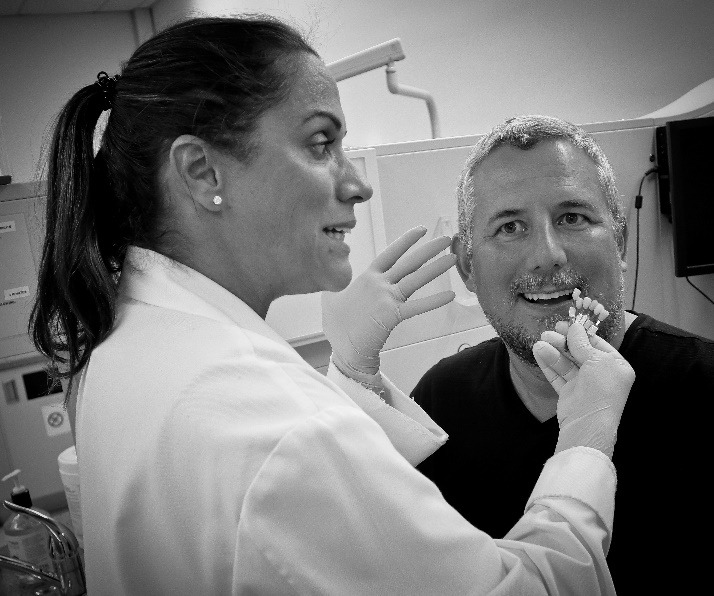
Traditional approach for shade determination.

The process of shade selection is somewhat subjective (Figure 1), with no consensus on protocol.^2^ Instrumentation has been developed to assist with the procedure, with limited success, variability in results and cost hindrance.^7-8^ In addition, shade selection has focused on tooth color, with little to no investigation into the pink shade selection to mimic the soft tissues.^9^

This research investigation aimed to develop a novel software application, coupled with peripherals, to provide an accurate, objective, and systematic approach to shade determination for teeth, soft tissues, and dental materials. The research is an extension of technology that has been used in photography and cinematography, with a specific dental application that provides a predictable and successful workflow.

## Methods

**Figure 2:**
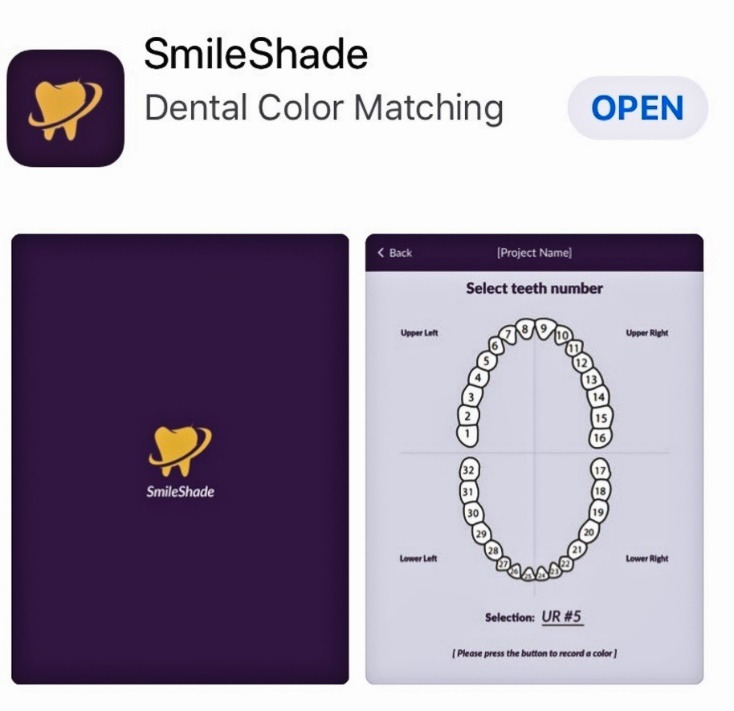
Smile Shade IOS app provides an alternative approach.

An IOS software application was developed, termed Smile Shade (Figure 2), to facilitate a simple, accurate, and systematic approach for dental shade determination. The application wirelessly pairs with a color scanning device and provides a seamless and stepwise workflow for the determination of dental shade.

### Functionality

**Figure 3:**
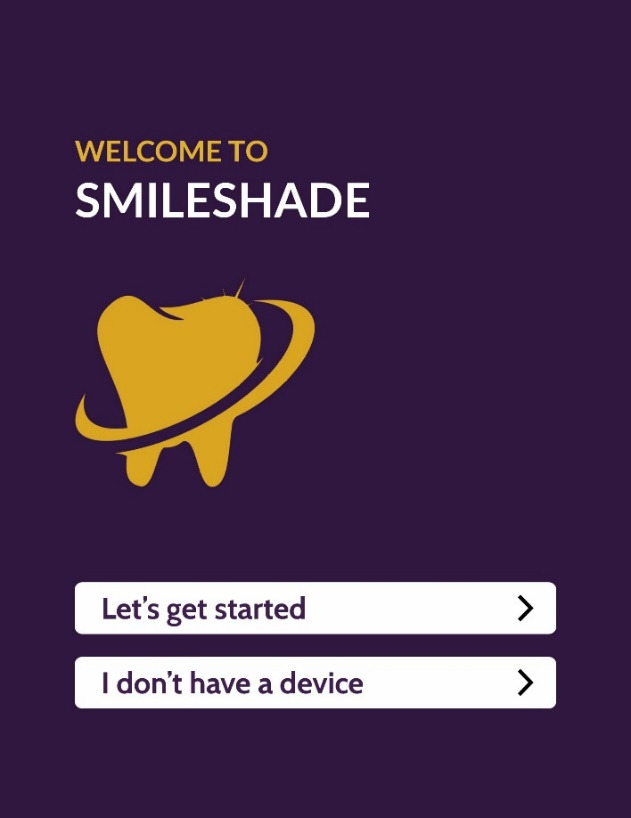
The opening page of the Smile Shade app.

**Figure 4:**
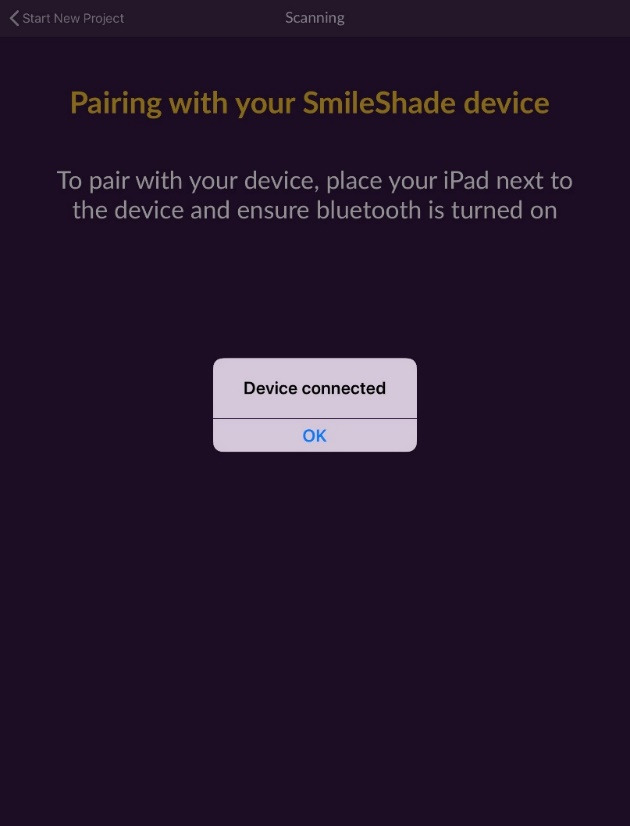
Wireless Bluetooth pairing of the app with the digitizer.

**Figure 5:**
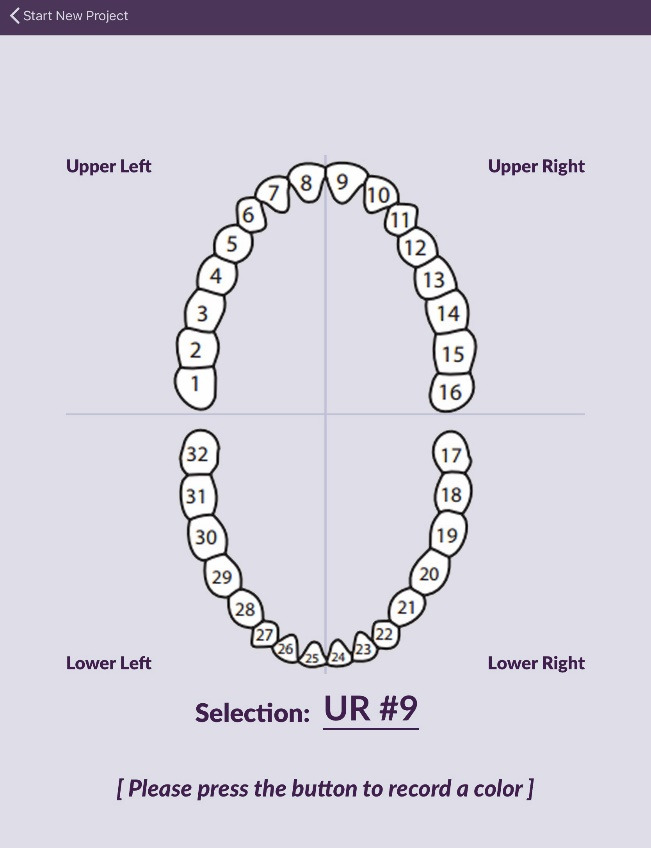
Tooth selection for shade determination.

**Figure 6:**
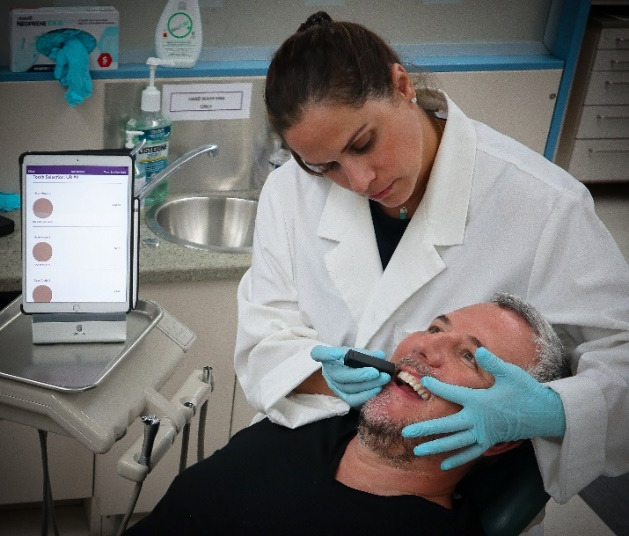
Clinical workflow of Smile Shade determination.

**Figure 7:**
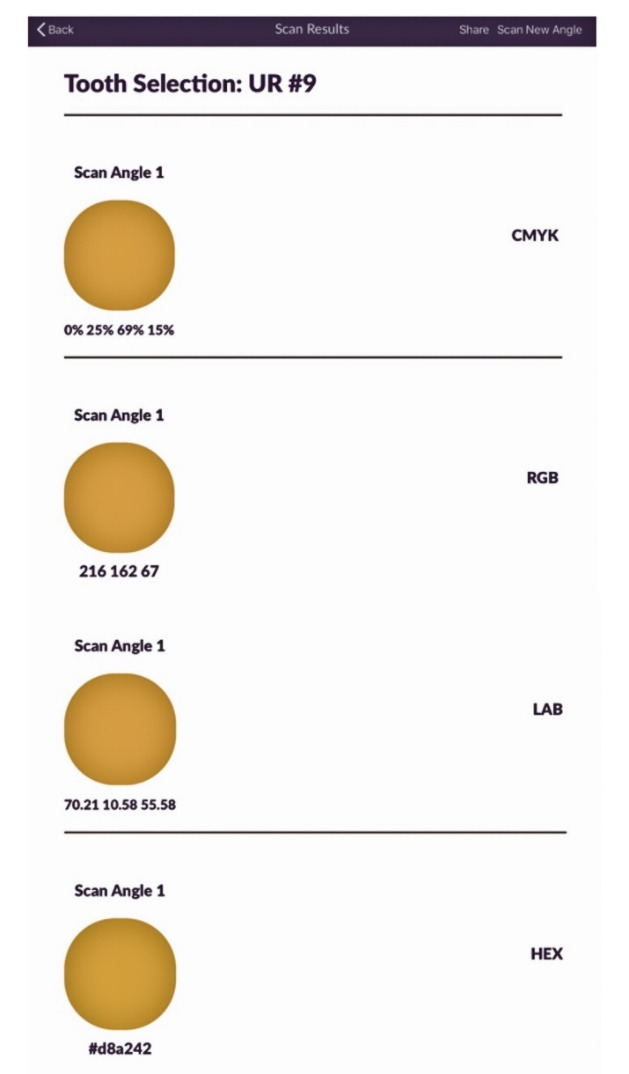
Shade determination and communication.

**Figure 8:**
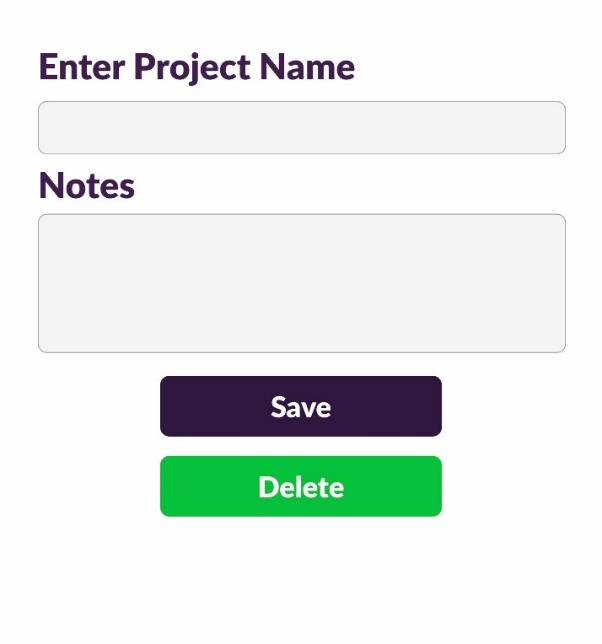
Digital workflow for shade determination.

The application (Figure 2) is only compatible with Apple’s iPad, and the app is free to download (Figure 3) but requires a color scanning device for operation (Figure 4). The device is available through the app by selecting the ‘I don’t have a device’ icon. Once the app has been downloaded, the operator opens the application and pairs it with the device through a wireless bluetooth connection (Figure 4). The operator then selects the tooth number requested for scanning (Figure 5). The operator then places the scanner to the surface that requires shade determination and presses the scan button on the device (Figure 6). The device requires approximately 2 seconds to generate a color determination of the material, and then the application creates a multiple screen display of the color (Figure 7). The operator may add a project name and appropriate notes and save the shade, or the file may be deleted (Figure 8). The file is stored on iCloud and may be downloaded or emailed for communication.

### Shade determination

Smile Shade functions on a high-dynamic-range micro-color sensor with automatic temperature control and inter-device repeatability of <1.0 ∆E. The determination of shade is completed through the evaluation of color based on CMYK, RGB, and LAB, which are different techniques of storing colors.

CMYK represents Cyan, Magenta, Yellow, and Key (Black).^10^ By using these colors, many other colors may be created. CMYK is typically used for tangible work, such as printing, and is considered “light” in nature.^10^

RGB represents red, green, and blue, and different colors can be created by adding many small pixels of red, green, and blue together.^10^ RGB is typically used to display digital work and is considered an industry standard to store color data digitally.^10^

LAB represents L for lightness, and A and B for the color dimensions (green and red, blue and yellow).^11,12^ The LAB color space is the most exact means of representation. This accuracy and portability make it suitable in several different industries, such as print, automotive, textiles, and plastics. Although the Lab color is the most exacting, it is not the most common. Lab color is usually converted to less accurate color, such as RGB and CMYK, as computer monitors and printers use either three or four colors to represent images.^12^

HEX was also included in the shade determination and is a syntax to encode the colors using hexadecimal values.^13^ The hex values may be used to code for RGB, CMYK, and LAB.^13^

## Discussion

The Smile Shade application was developed to provide a simple, systematic, and objective workflow for the determination of shade. The second part of the research investigation is currently underway to compare this novel application to the traditional shade tab approach commonly practiced at most dental schools. The data from the investigation will be available for publication in the late fall.

This novel approach to shade determination offers clinicians an alternative digital dentistry tool to provided simplicity and accuracy and a reduction in errors and remakes.

## Acknowledgements

The author acknowledges the assistance of J. Bae, the support of Red Square Labs and Palette, iTunes accessibility by Apple and the financial support provided by the Schulich Dentistry Internal Research Grant and Research Driven Inc.

## Author’s Contributions

N/A.

## Competing Interests

The author owns the intellectual property of Smile Shade.

## Funding

Financial support was provided by the Schulich Dentistry Internal Research Grant and Research Driven Incorporated.

## Ethical Issues

N/A.
